# Targeted Protein Degradation for Agricultural Applications: Rationale, Challenges, and Outlook

**DOI:** 10.1021/acsbiomedchemau.5c00111

**Published:** 2025-07-31

**Authors:** Denis Fourches, Joseph Pilotte, Brian E. Watts, Daniel J. Saltzberg, Robert M. Cicchillo

**Affiliations:** 688592 Oerth Bio LLC, 112 South Duke Street, Durham, North Carolina 27701, United States

**Keywords:** targeted protein degradation, crop protection, agriculture, ubiquitin proteasome system, E3 ubiquitin ligase, PROTAC

## Abstract

Farmers urgently need novel technologies to ensure global food security for the rapidly expanding population, yet crop protection has seen little innovation in decades. Mounting regulatory pressures, pest resistance, and environmental concerns are driving demand for novel sustainable solutions. Beyond traditional small molecule active ingredients, very few alternative modalities (*e.g*., peptides, RNAi, biopesticides) have reached the market. Meanwhile, targeted protein degradation (TPD) has emerged as a breakthrough modality for human therapeutics, with numerous proteolysis-targeting chimeras (PROTACs) and molecular glues (MGs) advancing through clinical trials. Those compounds induce potent and selective degradation of protein targets via the ubiquitin-proteasome system (UPS). Recently, PROTACs have been shown to function in both insect cells and in whole insect organisms, marking a pivotal step toward their use as next-generation crop protection solutions. In this perspective, we showcase the rationale, key challenges, and potential breadth of applications of targeted protein degraders for agricultural purposes. The TPD technology is a promising and potentially disruptive alternative to traditional small molecule inhibitors in agriculture.

## Graphical abstract

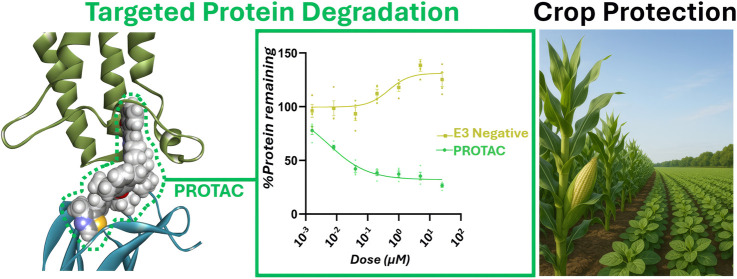

## Introduction

Modern agriculture has benefited greatly from advances in breeding, genetics, and chemical innovation, enabling farmers to effectively control weeds, insect pests, and fungal diseases—driving record crop yields.^1,2^ As global food demand increases, agriculture faces growing challenges from pest resistance, environmental constraints, and evolving regulations—all of which are contributing to longer development timelines, higher costs, and reduced availability of effective crop protection tools.^3–5^ While the 1980s ushered in several breakthrough classes of crop protection active ingredients—such as neonicotinoids (targeting insect nervous systems), strobilurins (fungal respiration inhibitors), pyrazole-based insecticides, and diamides (ryanodine receptor modulators)—innovation in new modes of action has slowed significantly.^6^ These chemistries have enabled lower application rates and supported integrated pest management (IPM) practices, but the pipeline of novel, development-ready solutions remains limited across the agrochemical industry.

In this context, alternatives to traditional small molecules are being revitalized, promising to transform agriculture with more innovative and sustainable solutions.^7^ For example, biological solutions comprise a range of technologies designed to provide more targeted, environmentally friendly, and sustainable pest and crop management. RNA interference (RNAi) technologies target specific genes in pests and pathogens to achieve precise control with minimal off-target effects. Peptide-based solutions leverage natural or engineered peptides to disrupt pest physiology or enhance plant defenses. Biopesticides, derived from microbes or plants, offer environmentally friendly alternatives to conventional synthetic products.^8^ Additionally, live microbial products are being deployed to enhance nutrient use efficiency and provide biocontrol benefits.^9^ Together, these technologies could represent a shift toward IPM strategies that prioritize ecological balance and long-term agricultural resilience, while also addressing challenges like resistance management and preservation of biodiversity. However, biologicals also face significant practical challenges, including high production/scalability costs resulting in potentially prohibitive prices for farmers, limited stability in field conditions leading to highly variable efficacy, and challenging delivery that can result in poor bioavailability and in turn mediocre effectiveness.^10^

Meanwhile, in drug discovery, targeted protein degradation (TPD) has emerged as a new modality, representing a paradigm shift in how disease-causing proteins are therapeutically targeted.^11^ Within the TPD field, proteolysis-targeting chimeras (PROTACs)^12^ represent the vast majority of compounds and have been shown to promote the degradation of many classes of proteins with high selectivity.^13^ PROTACs are heterobifunctional small molecules (Figure 1) that facilitate the formation of a ternary complex between an endogenous E3 ubiquitin ligase and a target protein-of-interest (POI) that enables the ubiquitination of target proteins. This mechanism harnesses the cell’s endogenous ubiquitin-proteasome system (UPS) for tagging and marking proteins for degradation. The UPS is a highly conserved system across eukaryotes, from yeast to mammals, and is distinct from prokaryotic degradation pathways, which are the subject of ongoing research.^14^ The UPS maintains cellular proteostasis by selectively degrading misfolded, damaged, and regulatory proteins, ensuring proper protein quality control and regulation of key signaling pathways.^15–17^ The PROTAC activity is driven by an event-based molecular mechanism that has important differences from traditional occupancy-based small molecule inhibitors. Yet untapped opportunities exist for degrading challenging protein targets that lack catalytic activity or defined active sites—such as scaffolding proteins and transcription factors—as well as for rescuing previously discarded targets with suboptimal inhibitors but adequate binding affinity. PROTACs also hold the potential to overcome resistance mechanisms by enabling removal of the target protein, even when only partial binding is achievable at mutated sites.^18–20^ Importantly, PROTACs operate catalytically—each molecule can trigger the degradation of multiple target proteins through iterative formation and dissociation of ternary complexes. This “catalytic effect” holds exciting promise to reduce dosages, extend duration of action, and lower the potential for off-target effects.^21–23^

**Figure 1:**
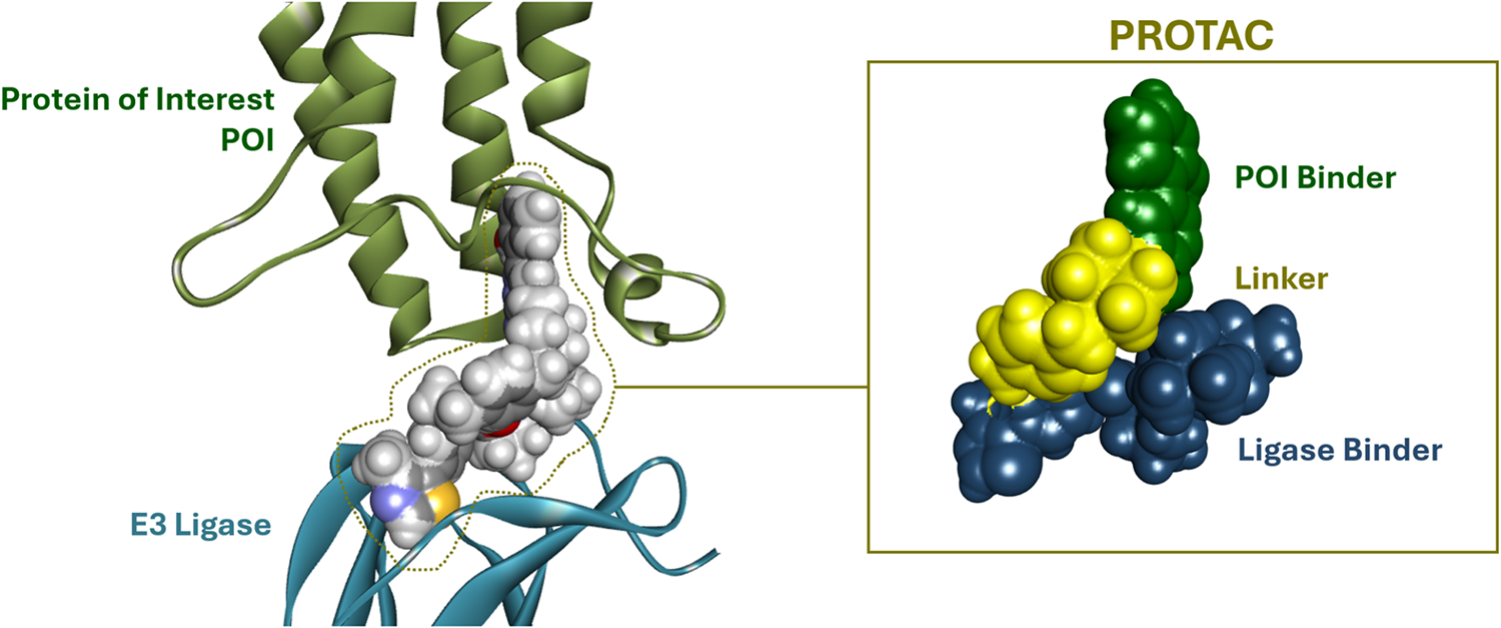
Overall architecture of a proteolysis-targeting chimera (PROTAC) facilitating the formation of a ternary complex. The PROTAC recruits an E3 ligase and brings in very close proximity a protein-of-interest (POI) promoting its ubiquitin tagging and then degradation via the proteasome. Example shown is from PDB: 6HR2, with the E3 ligase VHL and a BAF subunit.^82^

As TPD transforms healthcare, its extension into agriculture offers a promising new avenue for crop protection. The UPS machinery is functionally conserved across eukaryotes, underscoring the potentially broad utility of PROTACs for insect, weed, and fungal control in agriculture.^24,25^ Although knowledge of E3 ligases across kingdoms is advancing,^26,27^ the real-world applicability of PROTACs in agricultural systems has yet to be thoroughly explored. Depending on the context, degraders could for instance be designed for (i) eliminating critical pest targets, leading to lethality, or (ii) modulating plant immune or growth signaling pathways to enhance crop resilience. While traditional approaches to discovering bioactive small molecules for crop protection often rely on large chemical library screening with limited target knowledge, the emergence of rationally designed PROTACs could also enable selective degradation of well-defined biological targets and could thus represent a potential breakthrough in precision agrochemistry. Until recently, PROTACs had not been shown to function outside of mammalian cells.^28^

In 2019, Bayer and Arvinas launched Oerth Bio LLC, a joint venture created to merge Arvinas’ pioneering expertise in targeted protein degradation with Bayer’s leadership in crop science innovation. This collaboration was created to unlock the potential of PROTACs for agricultural applications and accelerate the discovery of novel crop protection solutions. Since its founding, Oerth Bio has delivered major breakthroughs, validating the first PROTAC degraders in both insects and plants, and establishing a new “target-first” paradigm for next-generation sustainable agriculture.

In this Perspective, we highlight the rationale, key challenges, and best practices for characterizing and assessing PROTAC degraders, along with the breadth of potential applications for protein degradation in agriculture. Together, these insights establish TPD as a promising alternative to traditional small-molecule inhibitors for crop protection.

## Recent Advancements in PROTACs: What Have We Learned from Human Therapeutics

### Increasing Diversity of E3 Ligases Identified as PROTAC-Compatible

One of the most significant breakthroughs in PROTAC research over the past decade has been the expansion of novel E3 ubiquitin ligases that were deorphanized with small molecule binders and subsequently proven to be useful for TPD.^29,30^ Among the 600+ known E3 ligases in humans, not all are actually PROTAC-compatible (many not inducing UPS degradation) and even less are PROTAC-enabled.^31,32^ Since its beginnings in early 2000s, PROTAC technology has relied heavily on cereblon (CRBN) and von Hippel–Lindau (VHL) due to the extensive information regarding their respective ligands, ubiquitous expression, high degradation potency (defined by very high *D*_max_ values and very low DC_50_ values), and breadth of protein classes targeted.^33,34^ More recently, several additional E3 ligases (*e.g*., MDM2, IAP/XIAP, KEAP1, DCAF1, DCAF11, DCAF15, DCAF16, RNF114, FEM1B, KLHDC2) have been successfully utilized for PROTAC-mediated degradation.^35–38^ Such an extended toolbox of PROTAC-compatible E3 ligases is extremely valuable, as each E3 ligase can have different structural characteristics, cellular localization, and expression patterns. These differences typically lead to variable engagement with targeted proteins, resulting in dramatically different levels of activity and promiscuity, and ultimately, utility for protein degradation. Therefore, when designing a PROTAC, selecting a specific E3 ligase can allow for precise targeting of a given protein located in specific tissues or cancer types in human therapeutics.^39^ Such precision would be extremely valuable for degrading specific targets in plants, insects and fungi.

However, despite the recent enablement of new E3 ligases, the overwhelming majority of PROTACs (and MGs) in clinical trials are still CRBN- and VHL-based as shown in Table 1. In that context, significant research has been devoted to further optimizing their recruiting ligands. For CRBN, a compendium of novel imide-based derivatives has been discovered, displaying improved stability, potency, and selectivity. The race for more orally bioavailable VHL-based PROTACs is still in progress with most efforts focused on modulating biophysical properties through linker modifications.^40^

**Table 1:** PROTACs Currently in Clinical Trials for Different Human Diseases^85^

target	E3 ligase	drug	administration	status
BCL-XL	VHL	DT2216	intravenous	phase I—completed
BTK	CRBN	AC676	oral	phase I
	CRBN	BGB-16673	oral	phase I
	CRBN	NX-5948	oral	phase I
	undisclosed	ABBV-101	oral	phase I
	CRBN	HSK-29116	oral	phase I
BTK, IKZF1/3	CRBN	NX-2127	oral	phase I
AR	undisclosed	AC176	oral	phase I—stopped
	CRBN	ARV-110	oral	phase I/II—completed
	CRBN	ARV-766	oral	phase I/II
	CRBN	CC-94676	oral	phase I
	undisclosed	HP518	oral	phase I
ER	Undisclosed	SIM0270	oral	phase I
	CRBN	ARV-471	oral	phase III
	undisclosed	AC682	oral	phase I—stopped
EGFR	undisclosed	HSK-40118	oral	phase I
BRD9	undisclosed	FHD-609	intravenous	phase I—stopped
	CRBN	CFT8634	oral	phase I/II—stopped
KRAS G12D	undisclosed	ASP-3082	intravenous	phase I
STAT3	undisclosed	KT-333	intravenous	phase I—completed
NTRK	CRBN	CG001419	oral	phase I/II
BRAF (V600E)	CRBN	CFT1946	oral	phase I/II
BRD4	CHAMP	RNK05047	intravenous	phase I/II
MDM2, p53	MDM2	KT-253	intravenous	phase I
IRAK4	CRBN	KT-413	intravenous	phase I

### Increasing Diversity of Target Proteins

The first generation of PROTACs has been largely devoted to targeting well-characterized proteins with known small-molecule binders, such as bromodomains, nuclear hormone receptors, and kinases.^37,41^ Even though many were originally in the realm of proof-of-concepts and/or tool compounds, several are now making their way through clinical trials (Table 1). With considerable advancements in molecular modeling, structural biology, and high-throughput screening, the PROTAC field has experienced a rapid expansion to broader and more diverse target proteins. For instance, there are several examples of potent degraders for nonenzymatic scaffolding proteins (*e.g*., FAK) or epigenetic regulators (*e.g*., HDAC).^42,43^ The PROTAC-DB database, a database of PROTAC structures and activity information collected from published papers, has grown from ∼1,600 PROTACs targeting 200 POIs in 2021 to more than ∼6,000 PROTACs targeting 400+ different POIs in 2025.^44^ PROTACs have also been designed to selectively target individual members of protein families. For example, selective degraders have been developed for SMARCA2 versus SMARCA4, or for HDAC3 versus HDAC6—demonstrating the ability to achieve high selectivity among highly homologous targets.^45–47^ Although small-molecule inhibitors can achieve paralog selectivity, PROTACs add another dimension by leveraging the choice of E3 ligase to enhance that level of selectivity. Importantly, the progress in linkerology,^48^ *i.e.*, the design and optimization of PROTAC linkers, has pushed the field to obtaining degraders for a given POI faster, especially when it comes to smaller rigid linkers. This optionality is particularly valuable for targeting pest proteins using nonselective ligands.^49^

## Why PROTACS Are Needed as a New Modality in Agriculture

### Introducing the PROTAC Modality, Not Just Another Mode-of-Action

In the field of agrochemistry, a mode of action (MoA) typically refers to a specific biochemical process by which a compound exerts its effect on a pest, pathogen, or weed. Historically, new agricultural products have been classified as novel based on their ability to target different enzymatic pathways or biological mechanisms compared to previously existing solutions. However, it is vital to differentiate a modality from a mode of action, that distinction becoming critical with the advent of PROTACs for crop protection and resilience.

Indeed, a modality defines the broader mechanism by which a compound interacts with its target, including whether it functions as an inhibitor, agonist, allosteric regulator, or, in the case of PROTACs, a protein degrader. While most agrochemicals developed over the past century have operated through competitive inhibition or receptor modulation (occupancy-based disruption of pathways), PROTACs represent an entirely different modality that harnesses the naturally occurring, cellular degradation machinery to precisely eliminate a target protein from a cell. This distinction is essential because, while a PROTAC might still act on a familiar inhibitor target (*e.g*., acetyl-coenzyme A carboxylase),^50^ that PROTAC does so in a fundamentally different way. Since the PROTAC brings the POI and the E3 ligase in proximity to form a ternary complex and enable the POI’s ubiquitination, this event-based mechanism depends on the PROTAC being able to bind and recruit both proteins. Interestingly, even ligands with relatively weak binding affinity (*K*_D_ ∼ 1 μM, compared to low nM or pM for classical inhibitors) can still form productive ternary complexes that lead to degradation.^51,52^ This suggests that a significant loss in binding affinity—such as a 100-fold reduction due to a resistance mutation—does not necessarily prevent PROTAC-mediated degradation. As a result, the PROTAC modality may be particularly well suited for agriculture, where resistance mutations in field populations are common.

As noted earlier, once a ternary complex dissociates, the PROTAC can engage another E3-ligase/target protein pair to form a new complex. Unlike traditional small-molecule inhibitors that act in a 1:1 stoichiometric fashion, PROTACs can be recycled, enabling them to function catalytically. The catalytic effect of a PROTAC can vary widely and is highly tunable—shaped by factors such as the choice of E3 ligase, the binding kinetics of both ligands, the linker architecture, and the overall cooperativity ratio (α = *K*_D_^binary^/*K*_D_^ternary^)^53^ for the associated ternary complex. In an agricultural setting, the catalytic nature of PROTACs has the potential to enable reduced application rates compared to conventional inhibitors—an important consideration for environmental stewardship and global registration. Finally, as with many small molecules, PROTACs can bind at various locations of the target protein, not solely in an orthosteric site.^12^ PROTACs that engage allosteric sites with high selectivity may offer a powerful strategy for targeting resistant mutants and achieving species selectivity by exploiting less-conserved regions of the target protein. Overall, PROTACs present a major opportunity for enhanced potency, durability, and the ability to overcome resistance mechanisms.

### Limited New MoAs: A Growing Crisis for Farmers

The past few decades have seen a striking lack of truly novel modes of action in herbicides, fungicides, and insecticides—contributing to a growing resistance crisis across major cropping systems. For herbicides, the last widely recognized innovation was the introduction of 4-hydroxyphenylpyruvate dioxygenase (HPPD) inhibitors in the 1980s.^54^ Only recently has a new mode of action emerged with the development of tetflupyrolimet, a dihydroorotate dehydrogenase (DHODH) inhibitor for weed control in rice, now classified as Herbicide Resistance Action Committee (HRAC) Group 28.^55^ Meanwhile, resistance to glyphosate, ALS inhibitors, and ACCase inhibitors in many weed species are on the rise.^56^ Many new products offerings are reformulations, mixtures, or combinations of existing active ingredients, rather than innovative solutions that provide a distinct mechanistic advantage. A similar trend is observed in insecticides and fungicides, where resistance to neonicotinoids, pyrethroids, and strobilurins has continued to spread due to the overuse of compounds with overlapping MoAs.^57,58^ The limited innovation in new MoAs increases the risk of widespread resistance, leaving farmers with fewer effective options and higher costs associated with managing resistant populations. This innovation gap highlights the urgent need for disruptive technologies like PROTACs to drive the next generation of agricultural solutions.

### Foundational Work in Pharmaceutics Has Set the Stage but Great Challenges Await

While PROTAC research in the pharmaceutical sector provides a strong foundation, translating this technology to agriculture requires addressing distinct challenges not encountered in human medicine, some of which include:

- Absence of PROTAC-ready E3 ubiquitin ligases in agriculture: compared to the large compendium of biological studies available for PROTAC-ready E3 ligases relevant for human therapeutics, there is very little known about the vast majority E3 ligases in agricultural species of choice (very often including their localization, expression level, or conservation from species to species within the same kingdom) and up to recently,^28^ no E3 ligase with demonstrated ability to work with a PROTAC.
- Bioavailability in plants and pests: Unlike pharmaceutical drugs, agrochemicals must be effective under field conditions, requiring stability in soil, changing environmental conditions (*e.g*., UV exposure, extreme temperature, rainfall), and efficient uptake in plant tissues or pest organisms. Formulations will need to be optimized for environmental resilience, ensuring that they remain efficacious but degrade in a controlled manner to prevent unwanted persistence.
- Species-specific targeting: Agricultural products must act across diverse biological kingdoms—including plants, fungi, and insects—each with distinct target proteins and proteasomal systems. Species- or kingdom-specific E3 ligase recruitment strategies will likely be required, necessitating a deeper understanding of non-mammalian proteomics to guide PROTAC design.
- Scalability and cost: As with conventional agrochemicals, the large-scale production of PROTACs will require cost-effective synthetic routes compatible with industrial manufacturing. However, agricultural applications must also meet the additional challenge of broad hectare deployment, where large-volume, low-cost production is critical for economic viability.
- Regulatory considerations: Unlike human therapeutics, which are regulated by agencies such as the FDA, agrochemicals are evaluated under a different set of standards by regulatory bodies including the US EPA, OECD, and EFSA. PROTAC-based products are expected to follow the same general regulatory pathways as conventional crop protection chemistry, with an emphasis on toxicology, environmental safety, and nontarget effects.

## From Concept to Crop: Translating PROTACs into Agricultural Solutions

The development of rationally designed PROTACs for agricultural applications requires solving key fundamental scientific questions (Figure 2), most of which come with the necessity of developing new computational predictive techniques and first-in-the-world characterization and degradation assays for diverse agriculture-relevant cell lines and species. As illustrated in Figures 2 and 3, the ideal PROTAC hit in agriculture has high degradation potency with high target selectivity, broad spectrum activity within the desired kingdom of life, good general bioavailability with minimal formulation, and no mammalian and pollinator toxicity. In that context, one of the major hurdles is that PROTAC discovery is still largely an empirical, “brute-force” process, requiring extensive screening and optimization to identify potent compounds. This lack of predictive design tools can make PROTAC development time-consuming and resource intensive. Additionally, the field remains highly CRBN-focused, with the vast majority of successful PROTACs relying on CRBN recruitment. While this has driven substantial progress, it also limits the diversity of degradation strategies available. Broadening the range of viable E3 ligases, particularly those specific to different organisms in agriculture, is crucial for expanding PROTAC applications beyond human therapeutics. Another significant challenge is bioavailability; many PROTACs suffer from poor membrane permeability, metabolic instability, and difficulties in crossing biological barriers. In agriculture, where environmental stability, plant/pest uptake, and broad-spectrum targeting are key considerations, addressing bioavailability issues will be essential for developing practical and effective PROTAC-based agrochemicals. Continued innovation in formulation strategies, ligand optimization, and delivery mechanisms are critical to overcoming these limitations and unlocking the full potential of PROTACs for both medicine and agriculture. In this section, we discuss the most significant hurdles in developing agricultural PROTACs and the strategies designed to address these challenges.

**Figure 2:**
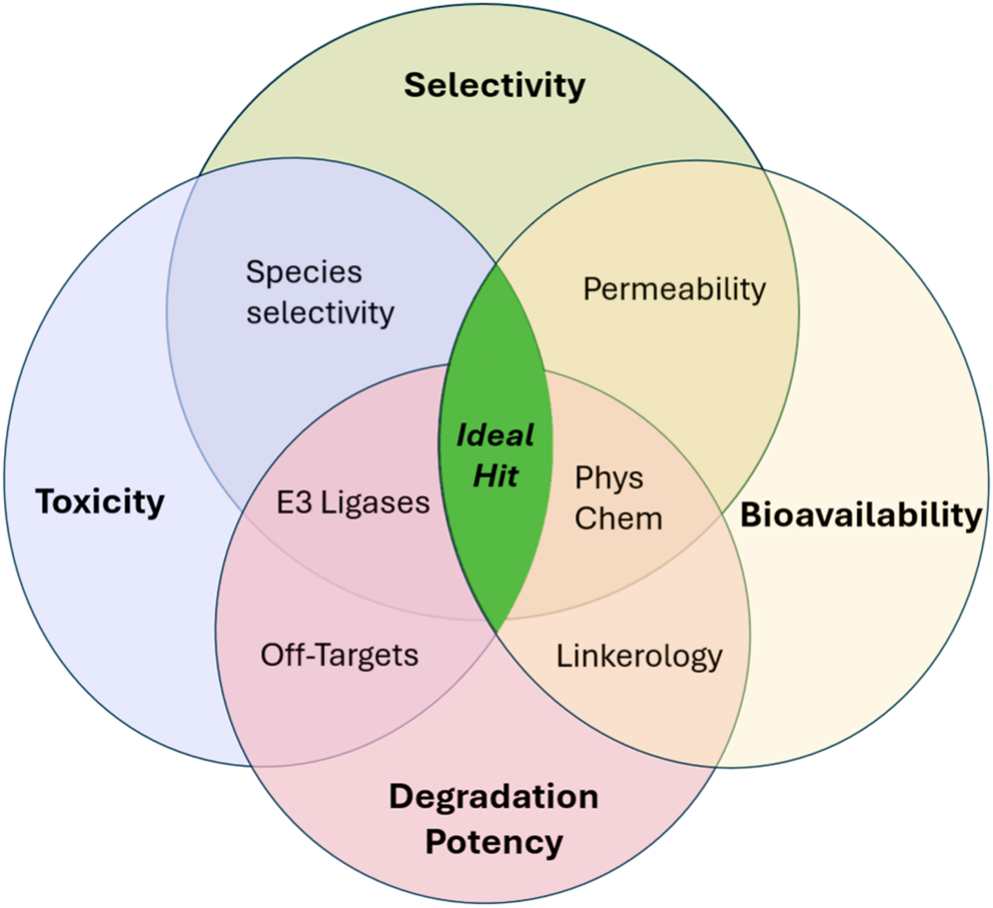
Venn diagram highlighting several key characteristics to consider when designing bioactive PROTACs. The ideal PROTAC hit in agriculture has high degradation potency with high target selectivity, broad spectrum activity within the desired kingdom of life, good general bioavailability with minimal formulation, and no mammalian and pollinator toxicity.

**Figure 3:**
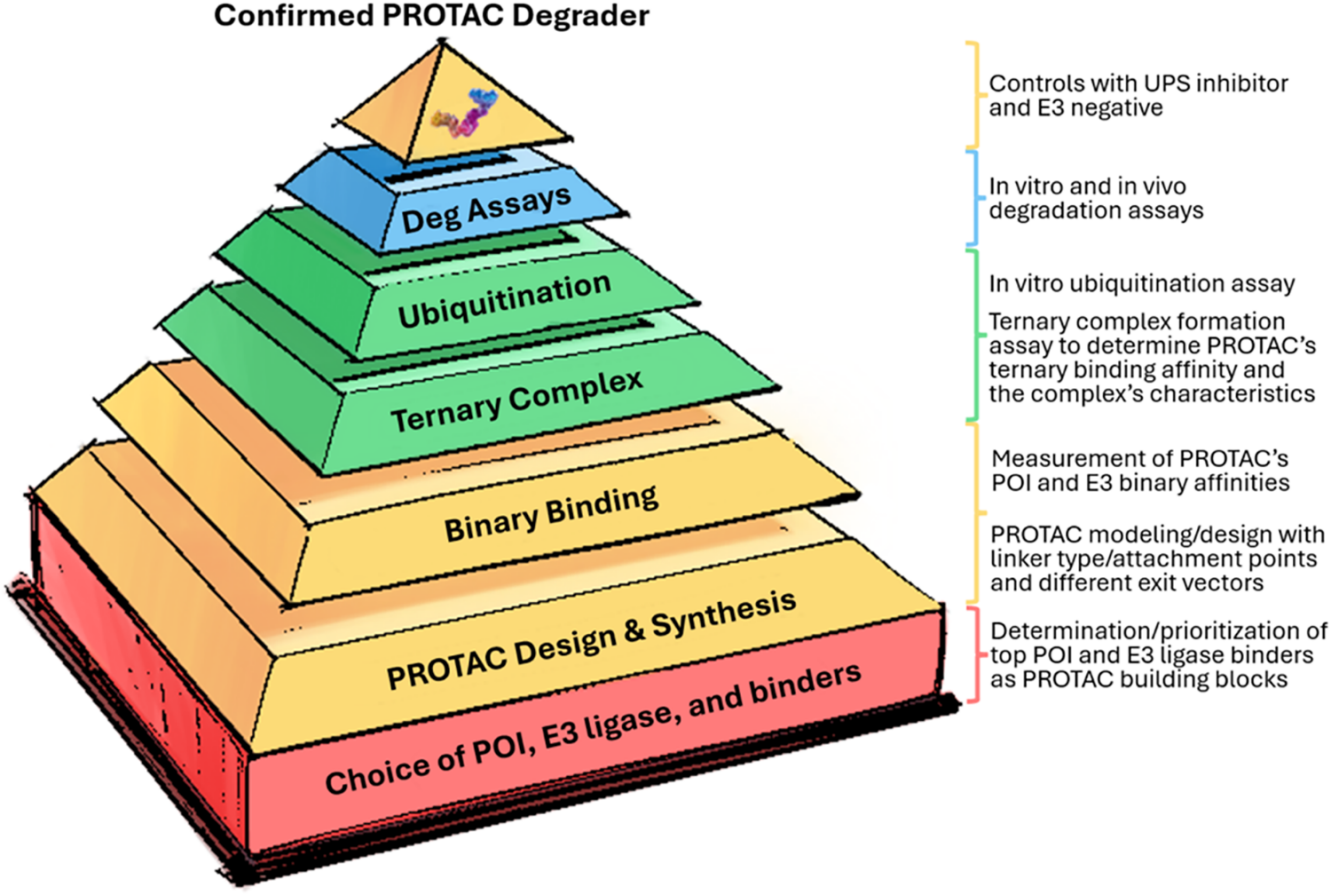
Key steps from PROTAC design to confirmed degradation activity. Each tier represents critical decision and validation stages in establishing binding, ternary complex formation, ubiquitination, and functional activity. This can also illustrate the chemistry SAR stack that is required to optimize PROTACs.

### Fundamental Scientific Challenges

Central to the success of PROTACs is the actual selection of target proteins, as not all proteins are amenable to degradation. Certain proteins like FKBP and BRD4 are considered highly degradable model proteins because a large compendium of PROTACs recruiting different E3 ligases and having simple linkers were shown to be potent against these targets.^59,60^ However, no *a priori* criteria exist to determine whether a given target protein is easily degradable via a PROTAC-mediated UPS mechanism. Schneider et al., introduced the notion of the PROTACABILITY score to identify targets specifically amenable to a PROTAC approach based largely on prior evidence of PROTAC-mediated degradation, known ubiquitination sites, and known small-molecule binders with available X-ray structures.^61^ In agriculture, potential targets lack this information, outside of some with a known small-molecule binder. Target selection and validation are critical to ensuring biological relevance and establishing the expected phenotype resulting from protein degradation. Key considerations in POI selection include cellular localization, the availability and properties of binding pockets, and the distribution of surface lysines. Other important factors are spatiotemporal expression, endogenous turnover kinetics (proteostasis or protein half-life), the presence of functionally redundant paralogs or isoforms, and oligomeric state.

Compounding this challenge is the scarcity of validated E3 ubiquitin ligases in relevant species, compared to the wealth of knowledge in human systems. The cross-species E3 orthology map (Figure 4) highlights both evolutionary conservation and divergence across major eukaryotic kingdoms. A conserved core of E3 ligases (depicted in slate gray) spans mammals, plants, fungi, and insects, indicating shared functional scaffolds that could support degrader design across taxa. Surrounding this core are numerous lineage-specific ligases, which reflect the unique evolutionary pressures and biological roles in each clade. These patterns underscore two complementary opportunities: the use of conserved ligases for broad applicability and the exploration of lineage-restricted E3s for species- or kingdom-specific selectivity in agricultural applications. Research driven by academic and industrial interest has yielded insights into several of these E3s in plants, insects, and fungi (Tables S1–S3). Certain E3 ligases are well characterized such as the plant F-box protein TIR1—long exploited by auxin herbicides and as a biochemical tool for protein degradation.^62^ For many of the hundreds of predicted E3 ligases, fundamental information regarding functional roles, expression patterns, degradation targets, and structural details is either vague or completely lacking. Moreover, there is a near-complete lack of tractable small-molecule ligands for E3 ligases in the agricultural space. However, conservation of ligases like VHL (in insects) and CRBN (in both insects and plants) has supported initial efforts to explore VHL-based PROTACs for insect control.^28^ Identifying and validating E3 ligases for agricultural use requires substantial investment in discovery, protein production, and ligand screening. The current lack of well-characterized and ligandable E3 ligases poses a significant challenge, limiting the near-term ability to design effective, species-specific degraders for agricultural systems. However, emerging technologies such as high-throughput screening, structural proteomics, and AI-guided ligand discovery offer promising avenues to accelerate the expansion of the E3 ligase toolbox.

**Figure 4:**
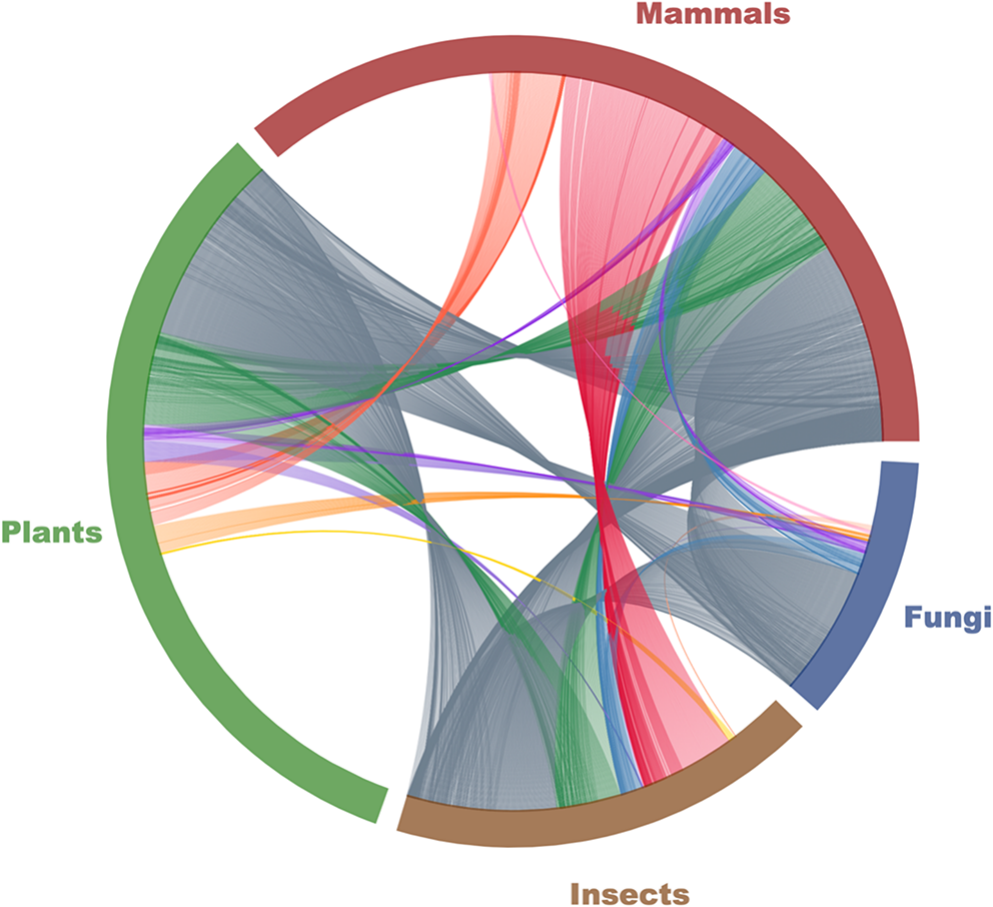
Orthology map of E3 ligases across Kingdoms of Life. Chord diagram of orthology among E3 ubiquitin ligases computed across representative species in four clades: mammals (*Homo sapiens*), plants (*Arabidopsis thaliana*), insects (*Drosophila melanogaster*) and fungi (*Botrytis cinerea*). Each arc represents all E3 ligases known/annotated in each species (iUCCD database, filtered at 80% sequence identity).^83^ Each chord connects two ligases belonging to the same homologous orthogroup, as computed by OrthoFinder;^84^ gray = conserved in all four clades; purple/blue/green = present in three; red/orange/yellow = shared by two; arc length lacking chords indicate clade-specific ligases.

At the molecular level, designing bifunctional degraders requires careful optimization of affinity (binary and ternary), linker length, flexibility, and spatial orientation. Unlike traditional inhibitors that rely on tight binding to an orthosteric or allosteric site, PROTACs must achieve binary interactions with both the target protein and the E3 ligase, while also promoting the formation of a stable, cooperative ternary complex. Fine-tuning this balance can be complex and time-consuming. However, molecular modeling approaches—such as machine learning-based QSARs, Generative AI, and 3D docking—can significantly accelerate design and optimization. It is well established that a PROTAC can form a ternary complex with an E3 ligase and a POI without inducing degradation—a phenomenon often referred to as a “non-productive” ternary complex.^63^ They are multiple reasons for this, with the most important ones being the wrong geometry of the ternary complex that prevents the transfer of ubiquitin’s, the absence of available lysine at the surface of the POI, or the lack of sufficient cooperativity between the two proteins once forming the ternary complex. Note that in the case of molecular glues, the ternary binding affinity is the most important as it determines the ability of the glue to bring the target and ligase together in a productive orientation that leads to ubiquitination and degradation.

### Physicochemical and Biological Challenges

PROTACs can be substantially larger than classical small-molecule inhibitors, typically ranging from 700 to 1300 g/mol, and often fall outside Lipinski’s Rule of Five (often referred as *bRo5*), contributing to challenges with solubility, cell permeability, and bioavailability in general.^64^ To address these issues, careful optimization of key physicochemical properties—such as molecular weight, lipophilicity logP, H bond acceptors/donors, and the actual solubility in biologically relevant environments like insect gut fluid or plant phloem—is essential. Interestingly, despite their size, some clinical-stage PROTACs lie surprisingly close to Lipinski’s thresholds,^65^ suggesting that well-balanced molecular design can yield compounds with favorable drug-like properties. Moreover, PROTACs’ chameleonicity^66^ in molecular properties is directly linked with the complex conformational space they can adopt in solution.

Achieving this balance requires a stepwise approach to structural optimization, often starting with the linker. However, improvements in physicochemical traits must be weighed against maintaining the spatial orientation and flexibility critical for effective ternary complex formation and degradation. Even minor modifications to enhance solubility can disrupt complex stability, rendering otherwise promising compounds inactive. Thus, PROTAC optimization should be treated as a multiparameter challenge (Figures 2 and 3), requiring the simultaneous balancing of chemical and biological properties, with *in vivo* degradation—and ultimately phenotypic outcomes—as the primary benchmarks for success.^48,67,68^

In parallel to physicochemical challenges, achieving selective degradation remains a critical consideration. Off-target effects have been reported with PROTACs, as typically assessed by mass spectrometry (MS)-based global proteomics.^69^ While highly selective degradation of the intended target is achievable, this requires a high degree of binary and ternary binding specificity. In some cases, PROTACs may bind related proteins without forming a stable ternary complex, preventing degradation. More commonly, degradation of additional proteins arises either from direct off-target effects—where structurally similar proteins are unintentionally degraded—or from indirect downstream consequences following degradation of a regulatory protein, such as a transcription factor or signaling molecule.^70,71^ Additionally, nonspecific E3 ligase recruitment, particularly by PROTACs incorporating hydrophobic tags or covalent handles, can potentially amplify off-target degradation across the proteome.^72^ Due to their catalytic nature, PROTACs may appear selective at early time points but can induce broader degradation profiles over time. Thorough time-course studies are therefore critical to distinguish direct off-target degradation from downstream biological consequences and to assess degradation-induced cytotoxicity, particularly in the context of safety and regulatory evaluations.

Finally, the *in vivo* chemical stability of PROTACs remains an emerging challenge with relatively limited data currently available in the public domain. As with traditional small molecules, metabolic transformations can compromise PROTAC efficacy by disrupting target binding, ligase recruitment, or ternary complex formation. However, metabolism can also be intentionally leveraged through prodrug strategies, where a modified, inactive form of the PROTAC is designed to release the active degrader in specific biological environments.^73^ Such approaches have already proven effective in agricultural applications, enhancing bioavailability, improving environmental stability, and enabling species-selective activation of small-molecule agrochemicals.^74^ As our understanding of PROTAC metabolism deepens, prodrug design considerations will become increasingly important for optimizing field performance, minimizing nontarget exposure, and supporting regulatory acceptance.

### Translational Challenges

Large-scale deployment of PROTACs in agriculture will depend on developing effective delivery systems tailored to the biology of target organisms. Compared to traditional small molecules, PROTACs often exhibit higher molecular weight, lower solubility, and greater structural complexity, requiring specialized formulation strategies. Complementary approaches such as nanoencapsulation, polymer-based carriers, and PROTAC-functionalized nanoparticles are being explored. Though the most scalable solutions remain to be determined, these advanced formulations aim at overcoming limitations in membrane permeability and systemic mobility, particularly in plant and insect systems.

Beyond chemistry-based improvements in solubility, stability, and uptake, delivery systems must also account for species-specific physiological and metabolic differences. Variability in E3 ligase sequence, expression patterns, proteasome activity, and cellular uptake mechanisms further complicate rational PROTAC design across diverse pests and crops. A deeper understanding of these biological differences will be critical to guide both target selection and optimization of PROTAC-based solutions.

Another critical translational consideration is large-scale manufacturing. While many of the challenges associated with PROTAC synthesis—such as synthetic complexity, cost, and the need for efficient conjugation of bifunctional molecules—are shared with pharmaceutical development, agricultural applications present additional constraints. Agrochemical production requires scalability and cost-efficiency, as it must support broad acreage field-deployable use cases where price sensitivity is high. Although PROTAC synthesis is well-established in pharma, adapting these processes for agrochemical manufacturing will require thoughtful optimization of chemistry and process design. This notably includes minimizing reliance on expensive reagents and chromatographic purification, reducing solvent use, and developing robust, high-throughput synthetic routes. Direct-to-biology platforms are highly effective for rapid hit generation and testing of PROTACs in early activity screens and structure–activity relationship (SAR) development. However, these must be complemented by scalable synthetic workflows to support commercial-scale production. Emerging technologies—such as biocatalysis and continuous flow chemistry—may help bridge this gap.^75,76^ Ultimately, scalable, cost-effective, and environmentally sustainable production will be essential to realize the commercial potential of PROTACs in agriculture.

Lastly, PROTACs designed for crop protection are expected to follow the same regulatory pathways as conventional agricultural inputs. However, their novel mechanism of action may require clarification within current frameworks to ensure an appropriate evaluation of safety, efficacy, and environmental impact. Early engagement with regulatory bodies and the generation of safety data aligned with established registration processes (e.g., OECD, US EPA, EFSA) will be important to streamline future approvals. For PROTACs aimed at improving crop resilience or efficiency, it is conceivable they could be classified as biostimulants—potentially benefiting from a faster path to market, particularly when using kingdom-specific E3 ligases.

## Best Practices and Accepted Standards in Degradation Assessments

Fully assessing and characterizing the degradation potency of a PROTAC presents a complex, multilayered challenge. Unlike classical enzyme inhibitors, degraders operate through a cascade of molecular events, requiring the recruitment of multiple proteins and the UPS. There is no single assay that tells the whole story for a given PROTAC, but rather a collection of key biochemical and biological assays that are needed to evaluate the multiple layers (Figure 3) of binding, degradation potency, selectivity, and bioavailability of a validated PROTAC. This section presents a set of best practices that are critical for rigorously validating the on-mechanism action of PROTACs in agricultural systems.

In early discovery, it is critical to demonstrate the binding affinity of a given PROTAC toward both the target protein and the E3 ligase. Efficient recruitment of these proteins is an important first step in the degradation pathway and in ensuring downstream biological effects are on-mechanism. Common techniques such as surface plasmon resonance (SPR), isothermal titration calorimetry (ITC), microscale thermophoresis (MST) and ligand displacement (LD) assays provide direct and quantitative measurements of ligand binding affinity. These methods are invaluable for generating SAR data, which can be used to further optimize ligands and vestigials for the design of new PROTACs. Therefore, the thorough biochemical evaluation of a PROTAC requires a suite of experimental approaches, going beyond the enzyme inhibition assays commonly employed for small molecule evaluation.^77^ Moreover, strong inhibition of the POI is neither the objective of PROTAC deployment nor an accurate reflection of the underlying binding properties.

In fact, binding kinetic parameters (*k*_on_/*k*_off_) can play an important role in distinguishing ternary complex behavior between different chemical scaffolds. The ternary complex formed by a PROTAC recruiting both an E3 ligase and a POI must be stable enough to facilitate the ubiquitination of the target. Indeed, longer-lived ternary complexes have been demonstrated to exhibit greater degradation in a VHL-bromodomain pairing.^78^ It is also important to measure and compare the binding affinity of the ligand alone with its corresponding vestigial (*i.e*., ligand augmented with a few atoms of the linkers) versus the full PROTAC. While linker attachment points on a ligand can be inferred from structural biology and modeling, it remains critical to test and validate these exit vectors. It is common to see a significant drop in binding affinity from the ligand to a vestigial, typically indicating the attachment point of the linker is not yet completely optimized. For this reason, investigating multiple attachment points for the linker is imperative in the design phase.

In some instances, multiple attachment points can be identified that facilitate strong binary and ternary interactions. In this scenario, synthetic feasibility and target selectivity may be important driving forces in deciding to which atom a linker should be attached. Overall, well-characterized ligands with extensive data regarding their binding affinity, inhibition potency, and structural interactions represent excellent starting points for PROTAC design. However, in the agricultural space, where historical ligands are often characterized by phenotypic outcomes rather than molecular binding profiles, this presents both a challenge and an opportunity for innovation.

The formation of a productive ternary complex—one that spatially enables ubiquitin transfer—remains a critical checkpoint. There are several methods for assessing the ternary complex formation *in vitro* including SPR, biolayer interferometry (BLI), and proximity-based fluorescence assays such as AlphaLISA and Förster resonance energy transfer (FRET). SPR and BLI are largely used for observing the kinetics and cooperativity of ternary complex formation via the direct interaction of ternary complex partners. Both SPR and BLI rely on protein immobilization, which can cause conformational restrictions and thus hinder ternary complex formation. To complement these approaches, AlphaLISA and FRET are solution-based assays that measure equilibrium binding parameters. Additionally, structural techniques like X-ray crystallography and cryo-electron microscopy (cryo-EM) provide direct insights into the spatial orientation of the ternary complex, offering valuable information for PROTAC refinement. Overall, integrating multiple orthogonal assays ensures a comprehensive understanding of both binary and ternary binding interactions prior to evaluating degradation or once degradation has been achieved and needs optimization.

Assessing the dependence of degradation on the UPS is another crucial step in validating a PROTAC’s mechanism of action. Beyond just the formation of a ternary complex, the PROTAC should facilitate a productive ternary complex.^79^ *In vitro*, gel-based methods have been developed to confirm ubiquitin transfer, a critical step prior to investigating engagement and degradation *in vivo*.^70^ *In vitro* ubiquitination (sometimes referred as IVQ or ivUb) methods can allow for direct observation of PROTAC-induced ubiquitination of a target protein in a dose-dependent manner (potentially allowing for a confirmation of the Hook effect with no ubiquitination at higher concentrations). The degree of ubiquitination and the concentrations at which it is most pronounced offer key indicators of ternary complex stability and productivity. While *in vitro* ubiquitination assay can be a useful checkpoint in PROTAC validation, it may not always be a suitable assay, as protein tags, E1/E2/E3 pairing, and post-translational modifications can influence ubiquitination activity.

Additionally, degradation should be rigorously evaluated and confirmed to depend on proteasomal activity. Quantification typically relies on global or targeted mass spectrometry (MS), Western blot (WB), or cell-based assays with HiBit-tagged proteins. Developing, validating, and optimizing these assays often requires multiple iterations. In the agricultural context, these challenges are amplified by poorly annotated proteomes, limited availability of validated antibodies, and the frequent use of small-molecule probes originally designed for mammalian targets, which may not cross-react in plant or pest species.

As previously mentioned, many of the general challenges in the TPD field also complicate assessments for agricultural applications. These include (i) the lack of antibodies for target detection, (ii) poor detectability of proteins by MS without extremely high-resolution capabilities, and (iii) the potential for biochemical tagging strategies to disrupt degradation mechanisms. In all cases, it is critical to demonstrate that observed degradation is on-mechanism and UPS dependent, rather than alternative protein turnover pathways such as lysosomal degradation or autophagy. This is typically confirmed using proteasome inhibitors (*e.g*., MG-132, bortezomib) or E1 ubiquitin-activating enzyme inhibitors, which should prevent PROTAC-induced degradation if the process is truly UPS-dependent. Similarly, knockdown or knockout of the recruited E3 ligase should abolish degradation, further verifying that the observed effects are mediated through the intended pathway. Additionally, synthesis of PROTACs with E3 negative control ligands (*e.g*., use of inactive enantiomers) can further demonstrate UPS dependence.

Another major consideration in PROTAC validation is off-target degradation, especially in open biological environments such as crops or pests, where off-target degradation could pose ecological risks. Off-target assessments should be conducted using quantitative proteomics approaches, such as MS-based global protein degradation profiling to comprehensively map degradation events. Such broad-spectrum proteomic profiling can assist in identifying liabilities early in the development cycle, informing both design refinement and future regulatory positioning.

Finally, as degradation is a fundamentally different mechanism from inhibition, careful differentiation between inhibition-based and degradation-based phenotypes is essential. While inhibitors act by transiently suppressing protein function, PROTACs exert their effects by removing the protein from the system. As a result, PROTACs may lead to unique phenotypic outcomes that are not immediately comparable to those observed with traditional inhibitors and vary from one species to another. Properly distinguishing these effects requires time-course studies that track protein degradation kinetics, cellular recovery after PROTAC washout, and functional assays that compare inhibition and degradation outcomes in a context-dependent manner.

We envision a strategic framework of best practices for PROTAC evaluation in agriculture—one that emphasizes mechanistic rigor, orthogonal validation across assay platforms, and translational relevance under field conditions. Notably, this framework mirrors the stringent evaluation standards already established in the pharmaceutical PROTAC space, underscoring the importance of robust, mechanism-based development for this modality. As agriculture faces increasing environmental and regulatory pressures, a disciplined yet adaptable approach will be essential to fully realize the potential of TPD. Standardizing these practices will not only enhance scientific reliability but also facilitate reproducibility, regulatory alignment, and the broader adoption of PROTACs as a transformative tool in crop protection.

## First Examples of PROTACs for Agricultural Applications

As previously highlighted, there is a very limited number of studies reporting on TPD for agricultural applications. Recently, a team including Oerth Bio and Bayer Crop Science scientists pioneered the use of small molecule PROTACs in insects, demonstrating the degradation of endogenous proteins in cells and larvae of fall armyworm (*Spodoptera frugiperda*), an agricultural pest of over 350 host plants that is responsible for annual crop losses estimated to be tens of billions globally.^28^ Specifically, the study established PROTAC-mediated degradative abilities of the *S. frugiperda* VHL (*sf*VHL) E3 ligase and its recruitment via a small molecule ligand. After identifying *S. frugiperda* homologues of proteins known to be degraded by VHL-based PROTACs in human cells, we showed that *sf*VHL can be recruited by PROTACs to efficiently degrade two insect targets, *sf*BRD and *sf*WDS (as shown in Figure 5). UPS inhibitors and E3-negative PROTAC controls successfully rescued that degradation, proving the UPS-mediated mechanism of action. The two targets sfWDS and sfBRD3 considered for that study were solely chosen as proof-of-concept and were not necessarily intended as insecticidal targets per se. Future studies will involve PROTACs designed to degrade known targets with higher insecticidal potential (*i.e.*, lethal phenotype) as well as previously untapped pesticidal targets considered undruggable with traditional small molecule inhibitors. Moreover, insect-specific ligase recruiters, appropriate formulations, and their associated insecticidal phenotypes will be pursued. Overall, this study represents the first demonstration of fully validated PROTACs functioning in a nonmammalian system.^28^

**Figure 5:**
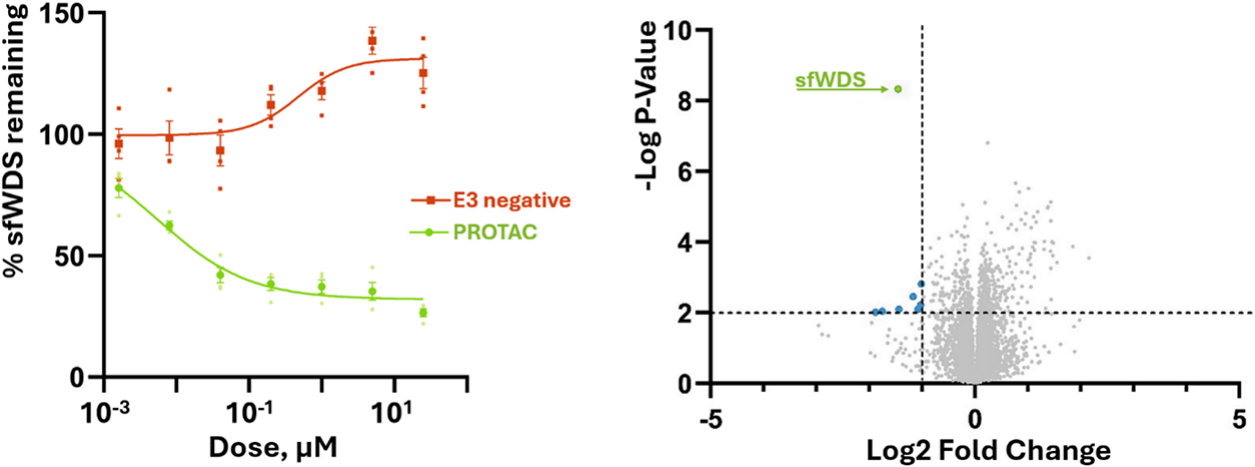
Example of confirmed *sf*WDS protein degradation in Sf9 cells: (left) Dose response of *sf*WDS degradation in Sf9 cells treated with DMSO, PROTAC (green) or E3 negative (red) for 24 h. *sf*WDS abundance was measured by targeted MS. (right) Global proteomic analysis of Sf9 cells treated with a *sf*WDS/VHL PROTAC represented as a volcano plot of Sf9 cell proteome resulting from treatment with 10 μM PROTAC for 4 h (*n* = 6 technical replicates). Each protein is indicated by a gray dot, *sf*WDS colored in green and other proteins with significant reductions in protein abundance (>50% decrease, *p* > 0.01) are colored blue. All data and detailed protocols are available in ref 28 as well as on the PRIDE repository (PXD062088).

To date, two other examples of PROTAC applications in agriculture include a granted patent (CN117756883A) and a recent publication by Xu et al.^80,100^ Both represent early attempts to adapt PROTAC technology for pest control, targeting essential enzymes, acetylcholinesterase (AChE) and acetyl-CoA carboxylase (ACCase). While these efforts introduce targeted protein degradation into the agrochemical space, their mechanistic validation remains limited compared to the standards established in the academic and pharmaceutical PROTAC literature. Each of the studies omit to demonstrate key biochemical evidence of ternary complex formation or confirmation of proteasome-dependent degradation. In the patent, only Western blotting was used to infer AChE degradation, without supporting data for E3 ligase engagement or UPS dependence. Likewise, the journal article omits several key validation benchmarks previously outlined. On-going attempts to reproduce these data, via evaluation of the reported CRBN–ACCase PROTACs at Oerth Bio, revealed poor target engagement and no degradation rescue upon UPS inhibitor treatment—potentially suggesting the observed reduction in ACCase levels are independent of PROTAC treatment. While these studies illustrate a growing interest in agricultural applications of PROTAC technology, they also underscore the need for more rigorous mechanistic validation to ensure full reproducibility.

## Outlook and Perspectives

TPD technology has the potential to significantly expand the chemical toolbox available for agricultural innovation. In this section, we explore how PROTACs could transform pest control by enabling the targeting of novel proteins or revitalizing previously drugged targets that have developed resistance. We also highlight unique advantages of this modality, including the flexibility to swap E3 ligases while degrading the same target, the potential to combine PROTACs with existing crop protection agents, and promising applications beyond pest controls such as their use in enhancing crop resilience.

### Degrading Completely New Targets

As discussed earlier, traditional small-molecule inhibitors typically act by tightly binding to an enzyme’s active site to block its catalytic function. However, many agriculturally relevant proteins—such as regulatory proteins, scaffolds, and transcription factors—lack defined enzymatic sites or are poorly inhibited by existing molecules, limiting their accessibility to conventional small-molecule approaches. These are precisely the types of targets where PROTACs offer a distinct advantage. Unlike inhibitors, PROTACs do not require equivalently high-affinity binding to a catalytic site; they can exploit shallow or noncatalytic pockets if the interaction is sufficient to promote ternary complex formation and degradation.^63^ This significantly expands the scope of druggable targets to include nonenzymatic and previously “undruggable” proteins. In this context, AI-driven prediction of 3D structures for these novel protein targets as well as discovery of cryptic binding pockets in known proteins can provide valuable starting points for PROTAC design.^81^

Realizing this potential will depend on identifying suitable ligands for these orphan protein targets, underscoring the importance of ligand discovery efforts such as DNA-encoded libraries (DELs), virtual screening, and high-throughput screening. With these tools, PROTACs could be used to modulate previously inaccessible biochemical pathways—including those involving transcription factors or scaffold proteins—that are highly specific to certain pest species or crop systems. In doing so, PROTACs expand the spectrum of tractable targets in agriculture and support the development of more selective and sustainable crop protection technologies with reduced risk to beneficial organisms and the broader environment.

### Addressing Resistance Mechanisms with PROTACs

Building on the ability of PROTACs to degrade nonenzymatic and previously inaccessible targets, their event-driven mechanism also offers a compelling strategy for overcoming resistance that has developed against conventional inhibitors. One of the most pressing threats in agriculture is the widespread rise of resistance to existing crop protection solutions. Pests, weeds, and pathogens rapidly evolve through mechanisms such as mutations in target proteins, enhanced metabolic detoxification, or upregulation of alternative pathways to bypass inhibited proteins. In this context, PROTACs offer a promising strategy to counter resistance.

As previously mentioned, PROTACs can tolerate weaker binding affinities to a protein target. Therefore, a residue mutation that reduces inhibitor binding from nanomolar to micromolar affinity may still allow for an effective degradation if the ternary complex is sufficiently cooperative and stable. Because PROTACs act catalytically and often require lower concentrations than inhibitors to achieve biological effects, they could rejuvenate previously abandoned or underperforming agrochemicals. Small-molecule ligands that were once discarded for insufficient potency could be repurposed into PROTACs, extending the utility of valuable chemical scaffolds. We envision that degradation-based approaches at large (*e.g.*, PROTACs, molecular glues, RIPTACs, AUTACs) will be particularly valuable for addressing herbicide-resistant weeds, fungicide-resistant pathogens, and insecticide-resistant pests—opening a new era of sustainable technologies.

### Importance of E3 Ligase Selection

Beyond target engagement, the versatility of PROTACs is further enhanced by the strategic selection of the E3 ligase, which plays a crucial role in determining species specificity, degradation efficiency, and overall safety profiles in agricultural settings. A defining advantage of PROTACs is their modular design, which allows for customization of both the linker and, most critically, the E3 ubiquitin ligase. Selecting the appropriate ligase is essential—not only for achieving efficient degradation of the target protein, but also for enabling selectivity across species, tissues, or cellular compartments. Because E3 ligases vary widely in their expression patterns, subcellular localization, and activity across organisms, ligase-POI pairing can dramatically influence precision and potency.

Even subtle changes—such as switching to a different ligand for the same E3 or to another recruiter for a different ligase—can produce distinct degradation profiles. This tunability allows PROTACs to be precisely tailored to agricultural applications. For example, a PROTAC that recruits a plant-specific E3 ligase will be inactive in insects or mammals, as the degradation mechanism requires the presence of that ligase to trigger ubiquitination. In its absence, the PROTAC remains inert—providing an inherent layer of species-level control.

In pharmaceutical development, it is well established that E3 ligase selection strongly affects PROTAC performance, including degradation efficiency, selectivity, and tissue specificity. Swapping the ligase recruiter—even while maintaining the same POI-binding ligand—can lead to profoundly different activity profiles. These principles translate directly to agriculture, where thoughtful ligase selection provides a powerful tool to control where and how a PROTAC functions. However, ligase switching is not trivial; it often requires reoptimizing linker composition and geometry, effectively resulting in a distinct molecule. This level of customization reinforces PROTACs as a highly adaptable and precise modality for sustainable crop protection.

While high selectivity is a hallmark of PROTACs, broad-spectrum efficacy remains an important goal for agricultural applications. Selectivity can be tuned not only through the design of the degrader itself, but also through thoughtful selection of both the target POI and the recruited E3 ligase. For example, targeting conserved or functionally essential proteins across a pest complex—paired with ligases that are broadly expressed in those species—could enable degraders with wider activity profiles. In parallel, multitarget PROTACs may further expand utility without sacrificing molecular precision. These strategies offer a path toward a “precision-with-coverage” paradigm, balancing the benefits of targeted degradation with broad-spectrum control.

### Strategic Integration of Degraders with Current and Future Active Ingredients

The agricultural value of degraders will ultimately depend on their ability to deliver practical advantages—whether through superior performance, resistance management, improved selectivity and/or spectrum, or the introduction of a novel chemical modality with small-molecule formulation compatibility. While PROTACs can function as stand-alone products, they will also likely complement traditional agrochemicals, extending product utility and intellectual property life, or restoring efficacy in resistance-challenged systems. Their precision and modularity make them well suited for their complete integration into modern pest and disease management programs, particularly as components of IPM strategies where selectivity, reduced inputs, and sustainable use patterns are increasingly prioritized.

Although targeted protein degraders are mechanistically distinct from conventional inhibitors or antagonists, it obviously remains to be demonstrated whether they can consistently deliver superior field-level performance. Head-to-head comparisons in relevant pest and disease models will be essential to justify their development. Nevertheless, degraders will offer several unique advantages: catalytic modes of action that can reduce required application rates, novel resistance-breaking potential, and improved specificity that can reduce off-target effects. These attributes make them an attractive complement to existing chemical classes and a strategic platform for next-generation trait development. Recognizing that development costs are nontrivial, early stage deployment may focus on high-value applications where degraders offer the most compelling return on investment.

Ultimately, PROTACs provide a completely novel chemical modality that complements conventional active ingredients, extends product utility and intellectual property life, and enables differentiated solutions that can equip farmers with more sustainable, targeted tools for pest management.

### Toward the Development of PROTACs for Crop Efficiency

Beyond crop protection, PROTACs hold strong potential as biostimulants and plant growth regulators, offering novel strategies to enhance crop efficiency and resilience. By selectively degrading regulatory proteins involved in stress responses, nutrient uptake, or growth signaling, PROTACs could improve plant adaptation to environmental challenges such as heat, drought, salinity, or soil imbalance. They could also be designed to fine-tune hormone signaling pathways (*e.g*., auxin, gibberellin, cytokinin), enabling optimized growth, increased biomass, and improved yield. While native plant hormone pathways such as auxin and jasmonate function through molecular glue-like mechanisms, rationally designed PROTACs offer a modular framework for targeting new proteins beyond these natural systems. It remains to be seen whether molecular glues or PROTACs will prove more effective in plant growth regulation; glues may offer inherent advantages in mimicking endogenous pathways, but their discovery is currently more empirical and less predictable, whereas PROTACs enable more deliberate and structure-guided rational design. Sprayable crop efficiency traits enabled by such technologies align with the broader vision for next-generation agrochemicals by 2050, and despite the challenges outlined in this Perspective, targeted protein degraders are well positioned to realize that goal.

## Supplementary Material



## Notes

Examples of E3 ligases identified in plants (S1), insects (S2), and fungi (S3) (Tables S1–S3); each table includes information on E3 ligase class, model organism, known biological functions, key substrates, and relevant literature references (PDF)
