# Supplementary material for: Targeted Protein Degradation for Agricultural Applications: Rationale, Challenges, and Outlook
Source: ACS Bio Med Chem Au. 2025 Jul 31;5(5):778–91. doi: 10.1021/acsbiomedchemau.5c00111 (PMC12531867; doi:10.1021/acsbiomedchemau.5c00111)
Supplement: Supplementary file 1 [file bg5c00111_si_001.pdf]

Targeted Protein Degradation for Agricultural Applications: Rationale, Challenges, and Outlook

Denis Fourches, Joseph Pilotte, Brian E. Watts, Daniel Saltzberg and Robert M. Cicchillo*

Oerth Bio LLC, 112 South Duke Street, DURHAM NC 27701, USA

*To whom correspondence should be sent; Email: rob.cicchillo@oerthbio.com

Supporting Information

Table of Contents

I. Title and Authors

II. Additional Data

Table S1. Examples of E3 ligases characterized in plants

Table S2. Examples of E3 ligases characterized in *D*. melanogaster

Table S3. Select E3 ligases characterized in fungi

Table S1. Examples of E3 ligases characterized in *A. thaliana*.

Ligases	E3 Ligase Class	Model Organism	Functions	Key Substrates	References
**KEG**	RING	*A. thaliana*	ABA signaling, stress response, seedling development	ABI5, CIPK26	[82]
**MAX2**	CRL1	*A. thaliana*	Strigolactone signaling, karrikin signaling	SMXL proteins (SMAX1-like repressors)	[83]
**PRT1**	UBR-1-like	*A. thaliana*	N-degron-mediated proteolysis, stress response	RIN4	[84]
**TIR1**	CRL1	*A. thaliana*	Auxin signaling	Aux/IAA (auxin repressors),	[85,86]
**COI1**	CRL1	*A. thaliana*	Coronatine, Jasmonic acid signaling	JAZ (jasmonate repressors)	[87,88]
**PUB13**	U-Box	*A. thaliana*	Immune signaling, flowering time	ABI1, LYK5	[89–91]
**SINAT1**	RING	*A. thaliana*	Root development, stress response	ATG6, FREE1, VPS23A	[92,93]
**ATL31/ATL6**	RING	*A. thaliana*	Carbon/Nitrogen response	CPK28	[94]
**RGLG1/2**	RBR	*A. thaliana*	Abscisic acid, drought stress response	PP2CA, NPR1 (immune signaling)	[95]
**COP1**	RING	*A. thaliana*	Photomorphogenesis, circadian clock	HY5, HYH, LAF1, PCH1, PCHL	[96,97]
**CHIP**	U-box	*A. thaliana*	Chloroplast development and low temperature response	PP2A, FtsH1, FtsH2	[98,99]

Table S2. Examples of E3 ligases characterized in *D*. melanogaster

Ligases	Class	Model Organism	Function	Key Substrates	References
**DIAP1**	RING	*D. melanogaster*	Apoptosis inhibition	DRONC	[100,101]
**Slimb**	CRL1	*D. melanogaster*	Wnt signaling, circadian rhythms	PER	[102,103]
**KEAP1**	CRL3	*D. melanogaster*	Oxidative stress response	Nrf2	[104]
**Parkin**	RBR	*D. melanogaster*	Mitochondrial quality control	Peanut, septin1	[105]
**BRWD3**	CRL4	*D. melanogaster*	Chromatin regulation	dCRY, KDM5	[106,107]

Table S3. Select E3 ligases characterized in fungi

Ligase	Class	Model Organism	Functions	Key Substrates	References
**Rsp5**	HECT	*S. cerevisiae*	Endocytosis, stress response, metabolism	PPxY motif, ROG3, LSB1, ROD1	[108,109]
**APC/C**	RING	*S. cerevisiae*	Mitotic progression, anaphase control	Mitotic Cyclins, securin	[110]
**Dma1/Dma2**	RING	*S. cerevisiae*	Cytokinesis, septin regulation	Sid4, Vac17, Mmr1, Pcl1	[111–113]
**Ubr1**	UBR-box	*S. cerevisiae*	N-end rule degradation	Scc1, Roc1, Thermosensitive proteins	[114,115]
**Doa10**	RING	*S. cerevisiae*	ERAD	Sbh2, Mata2, misfolded proteins	[116,117]
**Listerin**	RING	*S. cerevisiae, N. crassa*	Ribosomal quality control	Stalled nascent peptides	[118,119]
